# Screening of Efficient Adjuvants for the RBD-Based Subunit Vaccine of SARS-CoV-2

**DOI:** 10.3390/vaccines11040713

**Published:** 2023-03-23

**Authors:** Juan Shi, Yu Zhao, Min Peng, Suyue Zhu, Yandan Wu, Ruixue Ji, Chuanlai Shen

**Affiliations:** Department of Microbiology and Immunology, Medical School of Southeast University, Nanjing 210009, China

**Keywords:** SARS-CoV-2, receptor-binding domain, vaccine, adjuvants

## Abstract

The variants of severe acute respiratory syndrome coronavirus 2 (SARS-CoV-2) are more transmissible, with a reduced sensitivity to vaccines targeting the original virus strain. Therefore, developing an effective vaccine against both the original SARS-CoV-2 strain and its variants is an urgent need. It is known that the receptor-binding domain (RBD) in the S protein of SARS-CoV-2 is an important vaccine target, but subunit vaccines usually have lower immunogenicity and efficacy. Thus, selecting appropriate adjuvants to enhance the immunogenicity of protein-based subunit vaccine antigens is necessary. Here, an RBD-Fc subunit vaccine of SARS-CoV-2 has been generated, followed by vaccination in B6 mice, and four adjuvant regimens were investigated, including aluminum salts (Alum) + 3-O-desacyl-4′-monophosphoryl lipid A (MPL), AddaVax, QS21 + MPL, and Imiquimod. The adjuvant potency was evaluated by comparing the elicited polyclonal antibodies titers with measuring binding to RBD and S protein in ELISA and Western blot analysis, and also the cross-neutralizing antibodies titers using a pseudovirus infection assay of hACE2-expressing 293T cells, with pseudoviruses expressing the S protein of the SARS-CoV-2 original strain and Delta strain. The presence of QS21 + MPL adjuvant induced stronger polyclonal antibody response and neutralization potency blocking the original strain and Delta strain, as compared with the non-adjuvant RBD-Fc group and other adjuvant groups. Meanwhile, Imiquimod even had a negative effect in inducing specific antibodies and cross-neutralizing antibody production as an adjuvant.

## 1. Introduction

Severe acute respiratory coronavirus-2 (SARS-CoV-2), which causes Coronavirus disease 2019 (COVID-19), has demonstrated increased disease severity and high transmissibility, posing huge threats to human health and to the global economy [1,2]. Since its emergence in December 2019, SARS-CoV-2 has undergone continuous mutations, which have led to diverse variants of concern (VOCs), such as the Omicron (B.1.1.529), Gamma (P.1), Beta (B.1.351), and Alpha (B.1.1.7) variants [3]. Currently, most of these variants, particularly the Omicron variants, are resistant to the neutralizing antibodies induced by the first-generation COVID-19 vaccines [4,5]. Thus, the development of vaccines with broad-spectrum activity against multiple SARS-CoV-2 strains is critically important to prevent the global spread of SARS-CoV-2.

SARS-CoV-2 belongs to the β-coronavirus type, and its major structural proteins include spike (S) protein, membrane protein, nucleocapsid protein, and envelope protein. Among them, S protein is the most important structural protein and plays a key role in virus infection and pathogenic process. The S protein of SARS-CoV-2 is a homologous trimer structure, and each monomer consists of two subunits, S1 and S2. SARS-CoV-2 binds to the host cell surface receptor angiotensin-converting enzyme 2 (ACE2) via the receptor-binding domain (RBD) in the S1 subunit, and then the S2 subunit mediates the virus to fuse with the cell membrane of the host cell, thus completing the virus invasion [5]. Therefore, the RBD of S protein is a key target for the development of vaccines and therapeutic antibodies for SARS-CoV-2 infection [6,7,8,9].

Currently, SARS-CoV-2 vaccines in use or entering phase III clinical trials include nucleic acid vaccines (DNA vaccine/mRNA vaccine), protein-based subunit vaccines, non-replicating virus vector vaccines, and virus-like particles and inactivated vaccines, in which the protein-based subunit vaccines developed based on the production of key viral proteins in vitro from yeast, bacteria, mammal, or insect cells have a wide application potential thanks to their advantages of no viral genetic material, high safety, and being easy to produce, store and transport. However, subunit vaccines usually have lower immunogenicity, although this can be improved by appropriate adjuvants [10,11,12]. Therefore, the selection of appropriate adjuvants is also an important part of the subunit vaccine development process by which the humoral and/or cellular immune responses could be enhanced and prolonged. Adjuvants in development or in use mainly include aluminum salts, oil emulsions, nonionic block copolymers, saponins, microparticles, polysaccharides, immune-stimulating complexes, liposomes, cytokines, and bacterial derivatives [13]. In the present study, the RBD-Fc fusion protein was generated as a SARS-CoV-2 subunit vaccine, and the adjuvant potency of aluminum salts (Alum)+3-O-desacyl-4′-monophosphoryl lipid A (MPL), AddaVax, QS21 + MPL, and Imiquimod was evaluated, respectively, in anticipation of screening out an adjuvant regimen that is able to motivate the RBD-based subunit vaccine to generate a vigorous immune response.

## 2. Materials and Methods

### 2.1. Mice and Ethical Approval

Female and male B6 mice at an age of 6 to 8 weeks were used in our experiments. They were purchased from the Comparative Medicine Center of Yangzhou University (Yangzhou, China) and randomly assigned to different groups. All experiments related to these mice were conducted in strict accordance with the Guidelines for the Care and Use of Laboratory Animals (Ministry of Science and Technology of China, 2006). The experimental procedures and animal welfare were approved by the Animal Ethics Committee of Southeast University. The health of the mice was monitored every day during the experiment. A mouse would be removed from the study if it was lame, paralyzed, had nerve issues, reluctance to move, growth abnormalities, was losing 15% weight, exhibiting abnormal behavior, or had body temp decrease.

### 2.2. Recombinant RBD-Fc Protein Preparation

A plasmid encoding the S protein of wild-type SARS-CoV-2 (GenBank accession number QHR63250.2) was used to amplify the RBD sequence (residues 331 to 524) through PCR, followed by fusion with the Fc fragment of human IgG at the C terminal as described previously [8,9]. The recombinant plasmid was extracted and transfected into HEK293F cells (Courtesy of Dr. Lanying Du, Institute for Biomedical Sciences, Georgia State University) in a polyethylenimine (PEI) (Sigma, St. Louis, MO, USA) transfection experiment. At 72 h after transfection, the associated proteins were purified from the cell culture supernatant using an nProtein A Sepharose 4 Fast Flow (GE Healthcare, Chicago, IL, USA), and the concentration was measured using a Nanodrop (Thermo Scientific, Waltham, MA, USA).

### 2.3. SDS-PAGE and Western Blot

The boiled and non-boiled SARS-CoV-2 RBD-Fc proteins were analyzed using SDS-PAGE and Western blots. Briefly, the boiled and non-boiled SARS-CoV-2 RBD-Fc proteins were separated using 10% Tris-glycine SDS-PAGE, and then stained directly with Coomassie brilliant blue or transferred to nitrocellulose membranes (BIO-RAD). After being blocked overnight at 4 °C with 5% non-fat milk in PBST (phosphate-buffered saline buffer containing 0.05% of Tween-20), the membranes were co-incubated for 2 h at 37 °C with the sera (1:5000, prepared previously in-house) derived from the mice immunized twice with a 4-week interval with the SARS-CoV-2 RBD-His protein (10 μg/mouse, Thermo Fisher Scientific, RP-87675), and the sera were confirmed to contain RBD-specific antibodies through ELISA before use. After three washes, the blots were incubated with horseradish peroxidase (HRP)-conjugated goat anti-mouse IgG (1:10,000, Abcam, Cambridge, UK) for 1 h at 37 °C. After three to five washes, ECL substrate reagents (GE Healthcare) and a ChemiDoc™ MP Imaging System (Bio-Rad, Hercules, CA, USA) were used to visualize the signals.

### 2.4. Mouse Immunization and Sample Collection

The mice were immunized with the RBD-Fc protein in the presence or absence of adjuvants. In short, the mice were intramuscularly immunized with SARS-CoV-2 RBD-Fc protein (10 μg/mouse) and one of the following adjuvant(s): Alum (500 μg/mouse, InvivoGen, San Diego, CA, USA) + MPL (10 μg/mouse, InvivoGen), AddaVax (50 μL/mouse, InvivoGen), QS21 (10 μg/mouse, Desert King International, San Diego, CA, USA) + MPL (10 μg/mouse, InvivoGen), and Imiquimod (20 μg/mouse, InvivoGen). The second dose was injected after 3 weeks with the same protein and adjuvant (5 mice in each group). Meanwhile, the mice injected with SARS-CoV-2 RBD-Fc protein or PBS only were included as controls. 10 days after the last immunization, the sera from mice in different groups were collected, and specific antibodies and cross-neutralizing antibodies were detected.

### 2.5. ELISA

Assays to detect the SARS-CoV-2 S protein-specific antibody responses and SARS-CoV-2 RBD-specific antibody responses in mice sera were performed. Briefly, the S protein or the RBD protein without Fc fragment (1 μg/mL) was incubated in ELISA plates overnight at 4 °C. After washing once with PBST by using the 96-well half-area plate washing program of a Microplate Washer (BioTek, Winooski, VT, USA), the ELISA plates were further incubated with 2% non-fat milk in PBST for 2 h at 37 °C, followed by washing once more with the Microplate Washer. Then, serially diluted sera (started at 1:90, three times dilution) from immunized mice were added to the plates and incubated for another 2 h at 37 °C. After washing using PBST four times, the plates were incubated with HRP-conjugated goat anti-mouse IgG (1:5000, Abcam) antibodies at 37 °C for 1 h and washed with PBST four times again. Finally, the reaction was visualized through the addition of 3,3′,5,5′-Tetramethylbenzidine (TMB) substrate (Sigma) and stopped through the addition of 1N H_2_SO_4_ (Sigma). The absorbance of each well at 450 nm was measured using a Cytation 7 Microplate Multi-Mode Reader and Gen5 software (BioTek Instruments). The A450 value after calibration was used to plot the curve of each serum with GraphPad Prism 9, and four times the mean value of the negative control was defined as the cut-off line. Finally, the maximum dilution time of sera to reach four times the mean value of the negative control was calculated and defined as the sera-specific antibody titer.

### 2.6. Generation of Wild-Type and Mutant SARS-CoV-2 Pseudoviruses and Neutralization Experiment

As in our recent description [7,8,9], by inserting the target DNA sequence into a pcDNA3.1/V5-His-TOPO vector (Thermo Fisher Scientific, Waltham, MA, USA), we constructed the recombinant plasmid expressing the S protein of the SARS-CoV-2 original strain (GenBank accession number QHR63250.2), and based on this plasmid, the plasmids expressing the S protein of Delta variants containing two mutations (L452R, T478K) were constructed by using a QuikChange Multi Site-Directed Mutagenesis Kit (Agilent Technologies, Santa Clara, CA, USA), and confirmed through sequencing analysis. To generate the SARS-CoV-2 pseudoviruses, the plasmid encoding the S protein of the original strain or Delta strain, pLenti-CMV-luciferase plasmid and PS-PAX2 plasmid (Addgene, Watertown, MA, USA), were co-transfected into HEK293T cells (CRL-3216; RRID: CVCL_0063, ATCC) in a PEI (Sigma) transfection experiment. Then, the culture medium of transfected 293T cells was replaced with fresh Dulbecco’s modified Eagle’s medium (DMEM) 6 h later. Pseudovirus-containing cell culture supernatants were collected, and the titers were detected at 72 h after transfection.

The SARS-CoV-2 original and Delta strain pseudoviruses were incubated with serially diluted mouse sera at 37 °C for 1 h and then added to 293T cells that express human angiotensin-converting enzyme 2 receptor (293T-hACE2, from BEI Resources as item NR-52511; https://www.beiresources.org/ (accessed on 5 January 2022)). After 72 h co-cultures, the cells were lysed in cell lysis buffer (Promega, Madison, WI, USA), transferred to 96-well white opaque plates, incubated with luciferase substrate (Promega), and assessed for relative luciferase activity using a Cytation 7 Microplate Multi-Mode Reader and Gen5 software (BioTek Instruments). The neutralizing antibody titer was defined as the sera dilution times by which the viral infectivity was neutralized by 50% (NT_50_) [14,15]. NT_50_ values were calculated in GraphPad Prism 9 by using non-linear regression.

### 2.7. Statistical Analysis

The data generated in this study were analyzed using GraphPad Prism 9 statistical software. *Ordinary one-way ANOVA* and *Tukey’s multiple comparisons test* were used to calculate statistical significance across vaccination groups. *p* < 0.05 was considered statistically significant.

## 3. Results

### 3.1. Characterization of RBD-Fc Protein of SARS-CoV-2

**Figure 1:**
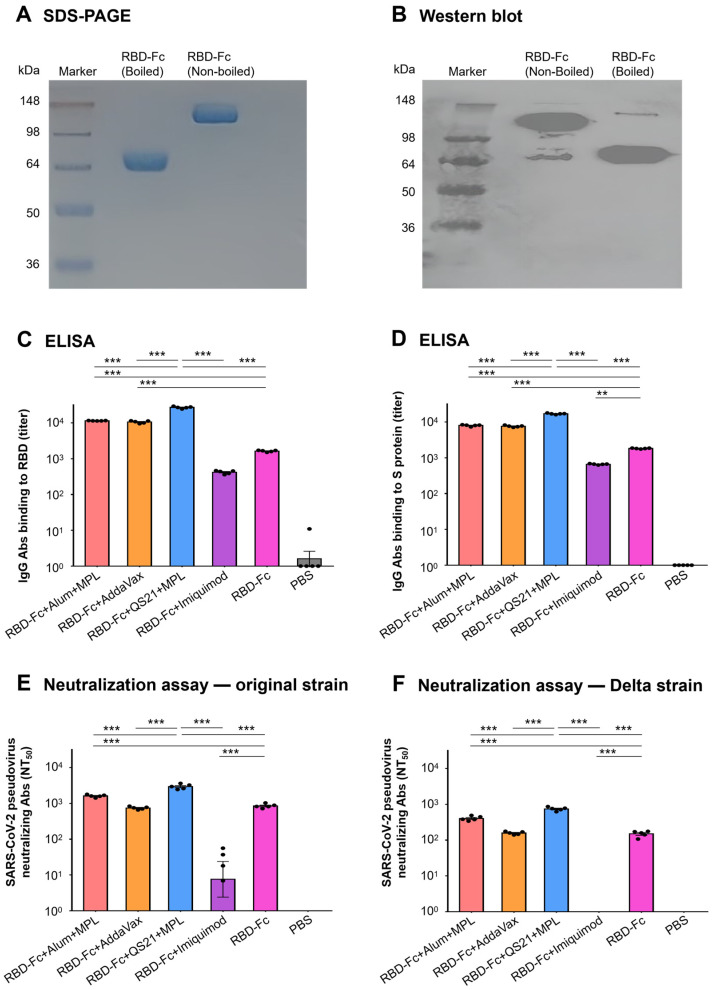
Design and evaluation of SARS-CoV-2 RBD-based subunit vaccine in the presence or absence of various adjuvants. SARS-CoV-2 RBD-Fc protein was expressed in HEK293F cells and purified from cell culture supernatants. The boiled and non-boiled SARS-CoV-2 RBD-Fc proteins were subjected to SDS-PAGE (**A**) followed by Western blot (**B**) with the sera from mice immunized with the wild-type RBD protein without Fc fragment of human IgG. Then, five B6 mice in each group were immunized with the protein twice, 3 weeks apart, in the presence or absence of various adjuvants. Ten days after final immunization, mice sera were collected and analyzed for SARS-CoV-2 RBD-specific antibody (**C**) and SARS-CoV-2 S protein-specific antibody (**D**) using ELISA. Furthermore, the neutralizing antibodies against SARS-CoV-2 original strain (**E**) and Delta strain (**F**) were also detected using pseudovirus, 293T-hACE2 cell line, and neutralization assay. NT_50_ was the dilution times of mice sera (neutralizing antibody titers) by which the pseudovirus infection in the 293T-hACE2 cells was neutralized by 50%. The data in the figure are expressed as mean ± SEM, and statistical significance was tested using *ordinary one-way ANOVA* and *Tukey’s multiple comparisons test*. Asterisks (*** and **) denote *p* values less than 0.001 and 0.01, respectively. The experiments were repeated twice with similar results.

The SARS-CoV-2 RBD-Fc protein was characterized using SDS-PAGE and Western blot analysis. SDS-PAGE showed that the molecule size of the boiled RBD-Fc protein was approximately 60 kDa and the non-boiled RBD-Fc protein had twice the molecular mass of the denatured (boiled) protein (Figure 1A), suggesting the dimer formation of the non-boiled RBD-Fc protein. In the Western blot assay, both the non-boiled RBD-Fc protein and boiled RBD-Fc proteins showed strong binding with the RBD-specific polyclonal antibodies in the sera from the mice immunized with the wild-type RBD protein without Fc fragment of human IgG (Figure 1B), indicating that the RBD-Fc protein maintained good antigenicity of the RBD fragment.

### 3.2. Screening of Adjuvants for the RBD-Fc Vaccine of SARS-CoV-2

#### 3.2.1. Inclusion of QS21 + MPL Adjuvant Significantly Improved the Antibody Responses

The in vivo immunogenicity of the RBD-Fc protein of SARS-CoV-2 was investigated in B6 mice in the presence or absence of various adjuvants. The mice were immunized with the RBD-Fc protein and one of the following adjuvant(s): Alum + MPL, AddaVax, QS21 + MPL, or Imiquimod, and followed by booster once. Immunization of control groups was performed in parallel. Then, mice sera were collected at 10 days after the final booster followed by the detection of RBD-specific and S protein-specific IgG antibodies using ELISA. As shown in Figure 1, when immunized without an adjuvant, the RBD-Fc protein induced specific IgG antibodies against the RBD protein with a mean titer of 1638.35 ± 38.50 (Figure 1C) as well as the S protein with a mean titer of 1823.24 ± 20.61 (Figure 1D), indicating the in vivo immunogenicity of the RBD-Fc protein. Statistical analysis results showed the presence of Alum + MPL (11,511.30 ± 61.10 and 8013.08 ± 186.29 binding to RBD and S protein, respectively), AddaVax (10,642.04 ± 373.94 and 7629.36 ± 184.46), or QS21 + MPL (27,117.99 ± 791.74 and 17,015.60 ± 297.32) adjuvants could significantly improve the antibody response with higher mean antibody titers than that of the non-adjuvant RBD-Fc group (*p* < 0.001), in which the presence of QS21 + MPL adjuvant induced the strongest antibody response, which was significantly higher than that of the non-adjuvant RBD-Fc group and other adjuvant groups (*p* < 0.001). In addition, the obvious decrease in mean antibody titer in the presence of Imiquimod adjuvant should not be ignored, as it may suggest that imiquimod is not a good choice as an adjuvant for the SARS-CoV-2 RBD-based subunit vaccine (*p* < 0.01). Meanwhile, in the control mice injected with PBS, only background levels of IgG antibodies were detected.

#### 3.2.2. Inclusion of QS21 + MPL Adjuvant Significantly Improved the Level of Cross-Neutralizing Antibodies

To assess whether the SARS-CoV-2 RBD-Fc protein induced high-level neutralizing antibodies in the presence or absence of various adjuvants, pseudovirus neutralization experiments were further performed by using pseudoviruses expressing the S protein of the original strain or Delta strain of SARS-CoV-2. Sera collected from B6 mice at 10 days after the second immunization were co-cultured with the pseudoviruses and then added to 293T-hACE2 cells. As shown in Figure 1E, when immunized without an adjuvant, the RBD-Fc protein induced neutralizing antibodies with a mean NT_50_ value of 853.85 ± 51.99, which can block the infection of original strain pseudoviruses. When combined with Alum + MPL or QS21 + MPL adjuvant, the neutralizing antibodies increased, with a mean NT_50_ value of 1617.78 ± 60.65 or 2976.43 ± 212.77, which are clearly higher than that of the RBD-Fc group (*p* < 0.001). Similar to the results of the specific antibodies, the NT_50_ value was highest when QS21 + MPL was used as adjuvant (*p* < 0.001). A similar phenomenon and trend can also be found in Figure 1F. The RBD-Fc protein from the original strain of SARS-CoV-2 also induced neutralizing antibodies against the Delta strain pseudoviruses, but the antibody level (mean NT_50_ of 151.00 ± 12.50) was much lower than the antibody against the original strain (mean NT_50_ of 853.85 ± 51.99). Alum + MPL and QS21 + MPL adjuvant also facilitated the RBD-Fc protein to induce neutralizing antibodies against the Delta strain pseudoviruses with a mean NT_50_ value of 407.32 ± 26.88 and 746.96 ± 39.65, which are significantly higher than that of the RBD-Fc group and lead to the same conclusion that the RBD-Fc protein combined with QS21 + MPL adjuvant produced the best neutralizing antibodies (*p* < 0.001). These data demonstrate that the RBD-Fc subunit vaccine can induce cross-neutralizing antibodies and the QS21 + MPL regimen possesses stronger adjuvanticity than other three adjuvants.

## 4. Discussion

The global outbreak of COVID-19 at the end of 2019 was quickly designated a public health emergency of international concern by the World Health Organization. With the continuous emergence of variants, the infectivity of SARS-CoV-2 is significantly enhanced and the transmission speed is significantly accelerated [16]. Therefore, there is an urgent need to find new prevention and treatment methods. It is known that protein-based subunit vaccines are safer than live attenuated or inactivated viral vaccines. Nevertheless, their immunogenicity and efficacy are low, and they need to be injected with appropriate adjuvants. Thus, continuous efforts are required to identify an adjuvant or adjuvant combination that could motivate the protein-based subunit vaccine to induce an enhanced immune protective response.

In this study, four adjuvant regimens were investigated, Alum + MPL, AddaVax, QS21 + MPL, and Imiquimod. As shown in the results, although the specific antibodies and neutralizing antibodies induced by the SARS-CoV-2 RBD-Fc protein in the presence of Alum + MPL adjuvant were significantly lower than in the group in presence of QS21 + MPL, it could still significantly improve the antibody titers when compared with the non-adjuvant group. As the most common adjuvant system, Alum + MPL is currently a component in two licensed vaccines against cervical cancer that contain oncogenic strains of human papillomavirus (HPV)-16 and HPV-18, and against hepatitis B virus [17,18,19,20]. In addition, previous studies showed that the ZIKV protein-based subunit vaccine had the best effect when Alum + MPL was used as the adjuvant, implying that Alum + MPL may also be suitable for other virus subunit vaccines such as non-ZIKV flaviviruses, SARS-CoV, MERS-CoV, and the SARS-CoV-2 virus [8,9,12]. Based on these existing applications and the results of our study, we believe that Alum + MPL is still a good adjuvant candidate for the SARS-CoV-2 protein subunit vaccine worthy of further exploration. MF59 is currently licensed in Europe for use as an adjuvant in influenza vaccines, and has been shown to enhance cellular (Th1) and humoral (Th2) immune responses to vaccines [21,22]. AddaVax, which is a squalene-based oil-in-water nano-emulsion, has a formulation similar to MF59, which indicates its potential to be used as an adjuvant. A previous study showed that when mice were immunized with a SARS-CoV-2 RBD-conjugated nanoparticle vaccine in the presence of AddaVax adjuvant, the resulting immunized mice sera were 8 to 120 times more active in neutralizing both pseudoviruses and the authentic virus than mice sera induced with monomeric RBD. Moreover, the sera of immunized mice more effectively blocked the binding of RBD to ACE2 in vitro, further confirming the promising immune effect [23]. With some regret, the study did not include a non-adjuvant control group, so it is hard to determine the role of adjuvants in improving immune effectiveness. The results of our experiments showed that although SARS-CoV-2 RBD-Fc protein combined with AddaVax adjuvant could exhibit significantly higher IgG antibody titers, the titers of neutralizing antibodies against the wild-type pseudovirus and Delta variant pseudovirus did not increase. Thus, the potential of AddaVax as an adjuvant for the SARS-CoV-2 RBD-based subunit vaccine needs further investigation. QS21 + MPL adjuvant has been used in enhancing specific immune responses to the antigen for selected candidate vaccines targeting malaria and herpes zoster [24,25]. It has been shown that QS21 + MPL adjuvant could significantly enhance both the cellular and humoral immune responses. A previous study has shown that the SARS-CoV-2 S1-based subunit vaccine could induce significantly stronger and sustained S1-specific IgG response and higher-level neutralizing antibody titer when using QS21 + MPL as an adjuvant compared to non-adjuvant regimens [26]. This conclusion was consistent with our experimental results, which also showed that the SARS-CoV-2 RBD-based subunit vaccine could induce the highest-level specific antibodies and cross-neutralizing antibodies against the original strain and Delta strain of SARS-CoV-2 in the presence of QS21 + MPL. These results all indicate that QS21 + MPL has great potential as an adjuvant for the SARS-CoV-2 protein-based subunit vaccine. Imiquimod is a guanosine derivative and agonist for Toll-like receptor (TLR)-7. It is an immune response modulator with potent antiviral and antitumor activity and is approved for the topical treatment of genital warts, basal cell carcinoma, and bladder cancer. It could activate dendritic cells (DCs) and B cells to induce cytokines optimal for Th1 cell immunity and antibody production, suggesting a potential role for Imiquimod as an adjuvant [27,28,29]. A study has also shown that Imiquimod could improve not only cellular immunity but also humoral immunity when used as an adjuvant for inactivated foot-and-mouth disease virus (FMDV) vaccines [30]. In addition, Imiquimod could effectively induce the production of virus-specific IgG and IgM when used as an adjuvant for inactive influenza virus vaccines, which are thought to be applicable for COVID-19 vaccine development [31,32]. More recently, it has also been demonstrated that the responses were shifted towards a Th1 phenotype, with marked enhancement of IgG2a, IgG2b, and CD8^+^ T cell responses and concomitant suppression of IgM and IgG1 responses when Imiquimod was used as an adjuvant for Ovalbumin [30]. The above-mentioned studies have different viruses, different types of vaccines, different immune pathways, and different methods to judge the effects, so the adjuvantability of Imiquimod cannot be conclusively determined. In our experiment, the sera-specific antibodies and cross-neutralizing antibodies of mice immunized with SARS-CoV-2 RBD-Fc protein in the presence of Imiquimod were significantly decreased compared to the non-adjuvant group. Notably, when measuring the titers of neutralizing antibodies against the Delta variant pseudovirus, the result of the imiquimod adjuvant group was no different from those of the PBS group. Therefore, based on these results, we do not consider Imiquimod suitable as an adjuvant for the development of the SARS-CoV-2 protein-based subunit vaccine.

As mentioned above, we evaluated the adjuvant potency by comparing the sera antibody titer induced by vaccination in mice, so as to determine the optimal adjuvant scheme. According to the results of this study, although these four adjuvant regimens could all induce effective specific antibodies and neutralizing antibodies in collaboration with the SARS-CoV-2 RBD-based subunit vaccine, their effects were quite different. Among them, Alum + MPL, AddaVax, and QS21 + MPL used as adjuvant all could improve the specific antibody response with higher mean antibody titers than that of the RBD-Fc group. In particular, the presence of QS21 + MPL adjuvant clearly improved the antibody response binding to the RBD and S protein, respectively, which was significantly higher than that of the non-adjuvant RBD-Fc group and other adjuvant groups. In tests to detect cross-neutralizing antibodies, the AddaVax adjuvant group did not show higher-level titers of neutralizing antibodies against the wild-type pseudovirus and Delta variant pseudovirus as it enhanced the specific antibodies. Fortunately, the cross-neutralizing antibodies increased with significantly higher titers than that of RBD-Fc group when Alum + MPL or QS21 + MPL was used as adjuvant. It is worth noting that the level of cross-neutralizing antibody in the QS21 + MPL adjuvant group was the highest and significantly higher than that of the non-adjuvant RBD-Fc group and other adjuvant groups. These results of neutralizing antibody test is consistent with those of specific antibody test. In addition, Imiquimod even had a negative effect in inducing specific antibody and cross-neutralizing antibody production as an adjuvant, which indicated that Imiquimod was not a good choice as an adjuvant for the SARS-CoV-2 RBD-based subunit vaccine.

## 5. Conclusions

In conclusion, the current data from immunized mice confirmed that the RBD-based subunit vaccine of SARS-CoV-2 can effectively induce antibody response and cross-neutralizing antibodies against the original strain and Delta stain of SARS-CoV-2, and more importantly, the vaccine was most effective when QS21 + MPL rather than Alum + MPL, AddaVax, or Imiquimod was used as adjuvant. Overall, this study identified QS21 + MPL adjuvant as the adjuvant of choice for SARS-CoV-2 RBD-based subunit vaccines, and has important implications for the subsequent development of the SARS-CoV-2 subunit vaccine.

## Data Availability

Data are contained within the article.

## Abbreviations

SARS-CoV-2	Severe acute respiratory syndrome coronavirus 2
RBD	Receptor-binding domain
S	Spike
Alum	Aluminum salts
MPL	3-O-desacyl-4′-monophosphoryl lipid A
COVID-19	Coronavirus disease 2019
VOCs	Variants of concern
HRP	Horseradish peroxidase
TMB	3,3′,5,5′-Tetramethylbenzidine
PEI	Polyetherimide
DMEM	Dulbecco’s modified Eagle’s medium
hACE2	Human angiotensin-converting enzyme 2
HPV	Human papillomavirus
TLR	Toll-like receptor
DCs	Dendritic cells
FMDV	Foot-and-mouth disease virus
